# Domestication of aromatic medicinal plants in Mexico: *Agastache* (Lamiaceae)—an ethnobotanical, morpho-physiological, and phytochemical analysis

**DOI:** 10.1186/s13002-020-00368-2

**Published:** 2020-05-01

**Authors:** Guadalupe Carrillo-Galván, Robert Bye, Luis E. Eguiarte, Sol Cristians, Pablo Pérez-López, Francisco Vergara-Silva, Mario Luna-Cavazos

**Affiliations:** 1 Posgrado en Ciencias Biológicas, Jardín Botánico, Instituto de Biología, Universidad Nacional Autónoma de México, Ciudad Universitaria, Ciudad de México, México; 2 Jardín Botánico, Instituto de Biología, Universidad Nacional Autónoma de México, Ciudad Universitaria, Ciudad de México, México; 3 Departamento de Ecología Evolutiva, Instituto de Ecología, Universidad Nacional Autónoma de México, Ciudad Universitaria, Ciudad de México, México; 4 Facultad de Ciencias, Universidad Nacional Autónoma de México, Ciudad Universitaria, Ciudad de México, México; 5 Posgrado en Botánica, Colegio de Posgraduados, Campus Montecillo, Texcoco, Estado de México México

**Keywords:** *Agastache*, Domestication, Ethnobotany, Aromatic medicinal plant

## Abstract

**Background:**

Most reports of domesticated plants that involve a domestication gradient or inter-specific hybridization in Mexico have focused on those used as food. This study provides knowledge about these processes in two aromatic medicinal plants, *Agastache mexicana* (Lamiaceae) and *A. m.* subsp. *xolocotziana*, widely used in Mexican traditional medicine for the treatment of gastrointestinal ailments and for their sedative effect. Different populations of *A*. *mexicana* along a gradient of domestication are found in the foothills of the Popocatepetl volcano of central Mexico, while in this same area the subsp. *xolocotziana* grows only in the cultivation, possibly a product of hybridization between *A*. *mexicana* and *Agastache palmeri*. This study links ethnobotanical, morpho-physiological, and phytochemical evidence to document the domestication of both taxa as well as elucidates the possible hybrid origin of the subsp. *xolocotziana*.

**Method:**

We analyze three groups of data derived from (1) 80 semi-structured interviews aimed at documenting the selection criteria related to the use and management of *A*. *mexicana*; (2) a cultivation experiment under homogeneous conditions, evaluating 21 floral, vegetative, and seed characters (that were important according to ethnobotanical information) in 97 plants corresponding to 13 populations of the taxa under study; and (3) the chemical profiles of the essential oils of these plants by means of a thin-layer chromatography.

**Results:**

By linking the three types of evidence, two evolutionary processes are distinguished: (1) *A*. *mexicana* occurs in the encouraged-cultivated phases of the domestication gradient and (2) *A*. *m.* subsp. *xolocotziana* may have originated through inbreeding depression or hybridization. These two cultivated plants show a domestication syndrome based upon organoleptic differentiation due to their dissimilar phytochemical composition and gigantism in flowers, seeds, and rhizomes (the last enhancing their asexual reproductive capacity). In addition to this, *A*. *mexicana* exhibits more intense floral pigmentation and foliar gigantism while subsp. *xolocotziana* presents floral albinism and partial seed sterility.

**Conclusion:**

Two divergent evolutionary processes are reported for the domestication of *A*. *mexicana* as a result of the intensification of its use and management. The selection processes of these plants have resulted in alternation of the organoleptic properties based upon the divergence of the phytochemical composition. Also, gigantism has been selected in culturally preferred plant parts and in correlated structures. The preceding characteristics reinforce the joint use of these plants in infusion in Mexican traditionalmedicine for the treatment of gastrointestinal diseases and for their sedative effects.

## Background

Domestication consists of evolutionary, dynamic, continuous, and multidirectional processes which lead the populations involved to a greater fitness through the selection that the human exerts on them, according to their use and management [1]. The domestication of plants in Mexico involves different degrees along a domestication gradient ranging with four major stages. The wild progenitors receive no conscious human attention, although they may be exploited. The tolerated individuals are allowed to persist usually in anthropogenic habitats contrary to other elements of the vegetation that are eliminated. The encouraged plants are promoted so as to favor the reproduction of the selected individuals with desirable characteristics and are subject to practices that improve to some degree the conditions in which they develop (e.g., protection against competitors and herbivores) [2–7]. Mexican examples include quelites [alaches (*Anoda cristata* (L.) Schltdl.) and quelite de agua *Amarantus retroflexus* L.)], bonnets (*Jacaratia mexicana* A. DC.), and hog-plums (*Spondias purpurea* L.). The domesticated plants have a greater fitness under cultivation and are propagated from vegetative parts, seeds, and/or transplants of complete individuals. Among the Mexican domesticates are maize (*Zea mays* L.), pumpkins (*Cucurbita pepo* L.), and beans (*Phaseolus vulgaris* L.) [2–7]. In other cases of domesticated plants such as prickly-pear cactus (*Opuntia ficus*-*indica* (L.) Mill.), guajes (*Leucaena* spp.), and agaves (*Agave* spp.) [8–10], inter-specific hybridization is an important mechanism of domestication and is currently of great interest [11]. The common suite of differential characteristics between cultivated domesticates and their ancestors (wild, tolerated, encouraged, or parental in the case of hybridization) is known as domestication syndrome, which includes gigantism in used parts and correlated structures, indicating its evolutionary process under domestication [1, 12].

Little is known about the domestication of medicinal plants in Mexico where between 3000 and 5000 native and introduced species are used by more than 69 indigenous peoples of the country. The main botanical families used in Mexican traditional medicine (MTM, hereafter) are Asteraceae, Lamiaceae, Solanaceae, in which many of their species are aromatic [13, 14].

Ethnopharmacological studies among the Popoluca (Veracruz), Mixe (Oaxaca), and Maya (Chiapas and Yucatán) indicate that aromatic organoleptic properties are a determinant for the consumption of plants for medicinal purposes ([15–19], respectively). These studies along with Geck et al. (2017a,b) confirm that odor and taste of plants are immersed in the culture, resulting in various organoleptic categories such as sweet, bitter, spicy, sour, fetid, among others [20, 21]. These characteristics are considered the main criteria in determining the appropriate treatment to alleviate given ailments. For example, among the Mixe people, sweet aromatic plants are preferred in the treatment of gastrointestinal ailments [16].

In addition, the plant’s morphology influences its preference for medicinal use. In the case of epazote (*Dysphania ambrosioides* (L.) Mosyakin and Clemants), leaf color, size, and shape, in addition to the organoleptic characteristics, are selection criteria of mestizo inhabitants with Mazatec ancestry of Santa María Tecomavaca, Oaxaca, to differentiate their employment of different forms as a condiment or an antiparasitic medication [22].

Genetic, environmental, and ecological variables, as well as the stage of plant’s development (seedling, plantlet, flowering or fruiting, etc.), influence their organoleptic characteristics, which are directly related to the chemical composition of their essential oil [23, 24]. Similarly, these variables contribute to the wide phenotypic variation within and between populations [24]. These variations in the domestication complex due to genetic selection need to be distinguished from the consequences of phenotypic plasticity in response to environmental variables [25]. It is important to demonstrate that management and selection lead to a morpho-physiological and organoleptic differentiation based upon genetic and phytochemical divergence between cultivated populations and their wild ancestors (the progenitor species or, in case of hybridization, the parental species). Several studies have suggested that the design of a common garden with homogeneous conditions reduces environmental variability. Thus, the expression of genetically based morphological and phytochemical differentiation between wild and cultivated populations allows one to identify selection criteria that humans have exerted on them [22, 26].

A good model to generate knowledge about the domestication process in aromatic medicinal plants of Mexico is Mexican hyssop belonging to the genus *Agastache* section *Brittonastrum* (Lamiaceae; Mentheae). Both *Agastache mexicana* (Kunth) Lint and Epling and *Agastache mexicana* subsp. *xolocotziana* Bye, Linares & Ramamoorthy are known in the MTM as “toronjil morado” and “toronjil blanco,” respectively, and are used together in infusions for their calming effect [27]. *A*. *mexicana* grows spontaneously and under cultivation throughout the Neovolcanense Province of central Mexico, especially in region of the Popocatepetl volcano, in the Ozumba Municipality, State of Mexico (Edomex), and in the Milpa Alta County, Mexico City (CDMX) [28, 29].

The subsp. *xolocotziana* only occurs in a cultivated state in central Mexico (CDMX, Edomex and Morelos). The absence of wild populations with white flowers, sterile pollen, and fruits, and vegetative reproduction via rhizomes, suggest that this taxon may be a product of hybridization [27]. Given the proximity of allopatric populations and the capacity of humans to breach the geographical barrier with *A*. *mexicana* (pine-oak forest, between 2800 and 3200 m asl), *Agastache palmeri* (B.L. Rob.) Standl. (of southern Sierra Madre Orientalense, especially in pine forest of Hidalgo and Puebla, between 2900 and 3200 m asl) was proposed as the other putative parental species [27, 30].

The basis of this hypothesis is that this subspecies has (a) morphologically intermediate phenotype, a single report of a morphologically intermediate, sterile hybrid between *A*. *mexicana* and *A*. *palmeri* [30]; (b) reduction in sexual reproduction, low viability in pollen (30%) compared to *A*. *mexicana* and *A*. *palmeri* (80% in both cases) [30; personal communication R. Bye]; (c) asexual reproduction, its propagation is vegetative through rhizomes [27]; and (d) novel characters such as the presence of approximately three times more compounds in the essential oil of the subsp. *xolocotziana* (38 compounds) compared to those found in *A*. *mexicana* (11 compounds) [31].

Documenting domestication processes in aromatic medicinal plants elucidates their evolutionary dynamics and compares its syndrome with other groups of plants used for other purposes. Hence, we can also lay the groundwork for understanding the domestication process in this complex and diverse group, little studied from this perspective [25]. This information is also the basis for implementing conservation and management strategies for these fundamental therapeutic resources in the MTM [32].

The objectives of this report are (1) to provide ethnobotanical, morpho-physiological, and phytochemical evidence for the understanding of the domestication processes of *A*. *mexicana*, by comparing different populations found along the domestication gradient, and the subsp. *xolocotziana* contrasting it with the putative parents, and (2) to generate information about the possible hybrid origin of latter taxon. We expected (1) the existence of organoleptic, morpho-physiological, and phytochemical dissimilarities between the populations involved in the domestication gradient of *A*. *mexicana* and in the populations of *A*. *m.* subsp. *xolocotziana* with respect to their putative parents, and (2) a pattern of differentiation of characteristics that coincide with hybridization as a possible origin of subsp. *xolocotziana*.

## Materials and method

### Studied species

*Agastache mexicana* (purple Mexican hyssop) and its subsp. *xolocotziana* (white Mexican hyssop) are aromatic herbs important in MTM. They are known and used beyond their natural geographic range, grown under cultivation, and commercialized internationally [13, 27]. Ethnobotanical, taxonomic, and phytochemical studies support their taxonomic relationship. The typical *A*. *mexicana* has an anis odor, purple corolla, triangular leaves with serrate margin in the lower blade, and essential oils with 11 compounds (the most abundant being estragole and limonene). Its subsp. *xolocotziana* has a mentholated odor, white corolla with trichomes on its lower lip, lanceolate leaves and crenate margin, and essential oils with 38 compounds (the most abundant being pulegone and limonene) [27, 31, 33].

The first written mention of *A*. *mexicana* occurred in the sixteenth century (1552) when it was registered in *Libellus de medicinalibus indorum herbis*, one of the oldest manuscripts of Mexican medicinal plants (also known as Codex de la Cruz-Badiano), under the Nahuatl name of *tlalahuehuetl*; its sap was applied to wounds [34–36]. A decade later, Francisco Hernández, the physician of the Spanish King Philip II, recorded *tlalahoehoetl* in the treatment of gastrointestinal ailments, urinary problems, and ophthalmological disorders in central Mexico [27, 37]. On the other hand, the use of the subsp. *xolocotziana* was not recorded until the twentieth century (1939) in *Las Plantas Medicinales de México*, being reported to treat gastrointestinal ailments and as an anti-spasmodic [38]. Currently, both aromatic herbs combined together in an infusion are drunk for treating gastrointestinal, cardiovascular, menstrual, and nerve pains; the “toronjiles” are also used to combat insomnia, as a sedative, and in the treatment of culturally affiliated ailments such as “susto” or “espanto” [27, 39, 40].

Recent phytochemical and pharmacological studies report the analgesic and anti-inflammatory effect of the organic extract of both taxa [40–42]. Similar extracts of *A*. *mexicana* relax bronchial smooth muscles of the guinea pig (*Cavia* sp.) [43] while those of subsp. *xolocotziana* induce contractions [44]. Anticonvulsant effect of the extracts of both taxa along with *Dracocephalum moldavica* L., an introduced Lamiaceae known as “toronjil azul” (blue hyssop), has been documented [45]. These three toronjiles (purple, white, and blue) form part of the “ethnobotanical medicinal complex toronjil” [46].

### Study area

**Fig. 1:**
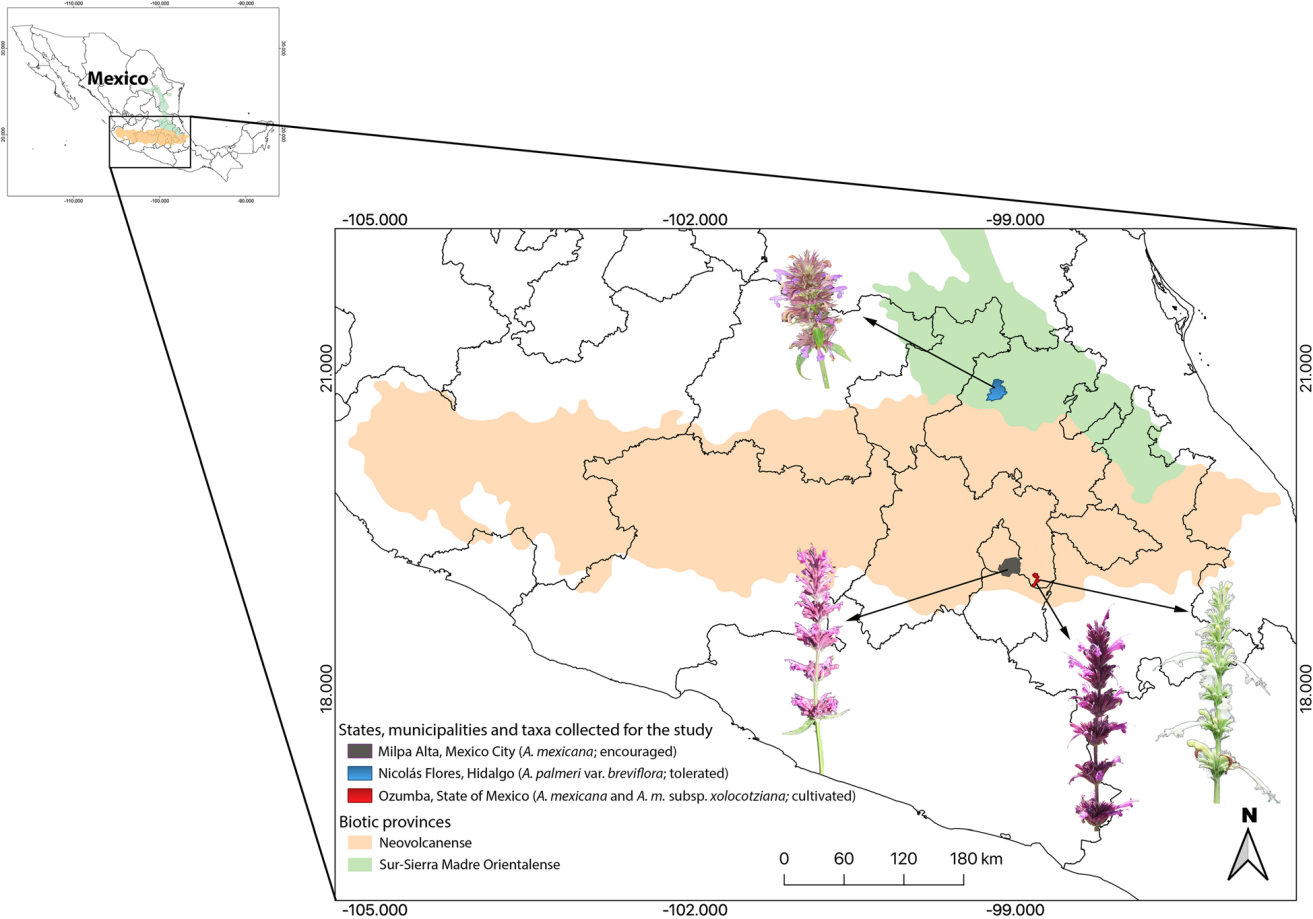
Geographic location, study sites and management categories of *A*. *mexicana*, *A. m.* subsp. *xolocotziana*, and *A*. *palmeri* in central Mexico. The images of the inflorescences illustrate the color variation according to the taxon and the management category

Based upon bibliographic searches, survey of specimens in the National Herbarium (MEXU–Herbario Nacional de México), and visits to markets in central Mexico, the following taxa and their management regime were detected: *A*. *mexicana*—cultivated and encouraged, *A*. *mexicana* subsp. *xolocotziana*—cultivated, and *A*. *palmeri*—tolerated. Three field study sites were selected (Fig. 1, Table 1):

**Table 1:** Taxa, management category, number of populations, number of individuals per population, total individuals, and sites considered in the morphological variation analysis. Initially, ten individuals were included in each population, however some did not survive

Taxa	Category	No. of populations	No. of ind. per population	Total no. of ind.	Locality
*A*. *mexicana*	Encouraged	4	9, 7, 7, 6	29	San Pablo Oztotepec, Milpa Alta, CDMX
	Cultivated	3	9, 8, 7	24	Santiago Mamalhuazuca, Ozumba, Edomex
*A.m.*subsp. *xolocotziana*	Cultivated	3	8, 7, 7	22	Santiago Mamalhuazuca, Ozumba, Edomex
*A*. *palmeri*	Tolerated	3	8, 7, 7	22	Puerto de Piedra, Nicolás Flores, Hgo.

1. Santiago Mamalhuazuca, Ozumba, Edomex. The municipality of Ozumba, located in the foothills of the western part of the Popocatepetl volcano (between 1800 and 2600 m asl; temperature ranges between 12 and 20 °C; precipitation between 800 and 1000 mm). The population is 27,207 inhabitants, the majority mestizos conserving a small Nahuatl indigenous sector (1.1%) [47, 48]. Cultivation and marketing of medicinal plants are major economic activities in this village. The principal species include *A*. *mexicana*, *Heterotheca inuloides* Cass., *Tagetes lucida* Cav., and *Justicia spicigera* Schltdl [29].. At this site, cultivated populations of both *A*. *mexicana* and the subsp. *xolocotziana* are found.

2. San Pablo Oztotepec, Milpa Alta, CDMX. Milpa Alta county is located in southern CDMX (average elevation 2420 m asl.), with a population of 130,582 persons, including the largest Nahuatl population in Mexico City [47]. The harvesting of medicinal plants, including *A*. *mexicana*, supplies the central medicinal plant market (Mercado Sonora) in downtown Mexico City [28]. In this locality, only encouraged populations of *A*. *mexicana* were found.

3. Puerto de Piedra, Nicolás Flores, Hidalgo. The municipality Nicolás Flores is located in the state of Hidalgo in the Sierra Madre Oriental mountain range, with 7031 inhabitants, where approximately half speak an indigenous language, mainly Otomi [47]. The vegetation is mainly pine-oak forests (elevations from 900 to 2800 m asl; precipitation from 800 to 1100 mm; temperature between 12 and 22 °C); part of this territory belongs to the Los Mármoles National Park. Also at this locality, tolerated populations of *A*. *palmeri* var. *breviflora* R.W. Sanders were managed.

### Ethnobotanical evidence

In the first two study sites, Ozumba, Edomex, and Milpa Alta, CDMX, visits were made every 2 months over a 2-year period. A total of 80 semi-structured interviews were conducted (40 at each site), in addition to botanical walks, participant observation and collection of plant material (seeds and complete plants) to document traditional knowledge, uses, and selection criteria (organoleptic and morpho-physiological characteristics) according to their use and management [16, 22, 49, 50]. The selection of the people for interviews was carried out by means of a “snowball” sampling [51]. The analysis of ethnobotanical data was carried out through summary statistics. In the third site, Nicolás Flores, Hidalgo, only seeds and complete plants of *A*. *palmeri* collected to document the two categories of evidence described below.

### Morpho-physiological evidence (under standardized conditions)

A common garden experiment was established within a greenhouse located in the Botanical Garden of the Institute of Biology (Instituto de Biología) of the National Autonomous University of Mexico (Universidad Nacional Autónoma de México) in Mexico City. Twenty-one morpho-physiological characters (vegetative, inflorescence, flowers, and seeds) were evaluated. These characters were based upon criteria for the use and management of these plants derived from the ethnobotanical inquiries (shown in Table 2, in addition to germination percentage). Because the harvest of the “toronjil” occurs when it blooms, data collection was carried out at the floral stage.

**Table 2:** Characters evaluated in the analysis of principal components and eigenvectors of the first and second principal components

Character	Units	PC1	PC2
Total height	cm	− **0**.**24450137**	− 0.25309168
Leaf area	cm^2^	− 0.20287308	− 0.19621089
Leaf color	pxs	0.10959718	0.012094649
Number of leaves	quantity	− 0.14272378	− 0.005930697
Number of teeth	quantity	− **0**.**25294183**	− 0.084785244
Number of stem nodes	quantity	− 0.12264778	− **0**.**457384999**
Inflorescence Length	cm	− 0.14389938	− 0.233460479
Number of inflorescence nodes	quantity	0.21010157	− 0.114973125
Number of flowers produced	quantity	− **0**.**2518971**	− 0.128068981
Style length	cm	**0**.**31203141**	− 0.20260069
Length of lower stamens	cm	**0**.**31459679**	− 0.225227546
Length of upper stamens	cm	**0.31311366**	− 0.224199747
Flower tube length	cm	**0**.**28171594**	− 0.293324118
Flower length	cm	**0**.**31435775**	− 0.223206556
Corolla color	pxs	0.11529446	**0**.**36229973**
Rhizome length	cm	0.11838912	**0**.**325060595**
Rhizome diameter	cm	0.01026666	0.043661684
Number of rhizome nodes	quantity	0.21010157	0.23809349
Seed length	cm	**0**.**27176266**	− 0.138544241
Seed diameter	cm	0.16784207	− 0.064307995

Bold values indicate the dominant characters in each component

Through a completely randomized design, four treatments were established consisting of three factors: (a) taxon, (b) degree of management, and (c) population. The treatments consisted of (1) *A*. *mexicana* + encouraged + Milpa Alta; (2) *A.mexicana* + cultivated + Ozumba, (3) *A*. *m.* subsp. *xolocotziana* + cultivated + Ozumba, and (4) *A*. *palmeri* + tolerated + Nicolás Flores. For each treatment, seeds were collected from three different populations in each location, except the first, where four populations were collected. The analysis of the morphological variation was obtained from a total of 13 populations and 97 individuals (Table 1). From each field population, voucher specimens were collected and deposited in the National Herbarium (MEXU).

### Statistical analysis of morpho-physiological characters

All analyses were performed with the Software R ver. 1.0.153 [52]. In order to document the pattern of grouping and discontinuities in the total variation, two analyses were made: (1) cluster analysis and (2) principal component analysis (PCA). For the first analysis, a matrix of population means was used, whose elements were standardized to mean = 0 and variance = 1; subsequently, a distance matrix was obtained using the square of the Euclidean distance. Cluster analysis was performed using the average distance method (unweighted pair group method using arithmetic mean—UPGMA) and represented by a dendrogram using a standardized average distance as weight. The total populations and characters were considered in this analysis.

The PCA was performed to analyze the relationship between the taxa under study and estimate the importance of the characters that discriminate among them. This analysis was carried out using a matrix that included 97 individuals and 20 morpho-physiological variables (the germination percentage variable was not considered for this analysis because it was obtained on the basis of populations rather than on individuals). Similarly, the elements of the matrix were standardized to mean = 0 and variance = 1. Subsequently, the matrix of correlations between the variables was generated, which served as the basis for calculating the characteristic values and vectors; next, the study units (individuals) were projected on the axes that represent the first two principal components.

In order to document significant differences between management categories and domestication trends in the 21 characters evaluated, we framed our analyses upon two questions. First, what are the differences between the encouraged and cultivated populations of *A*. *mexicana*? For this, Student’s *T* test was carried out considering a total of 53 individuals of *A*. *mexicana* (encouraged (29) and cultivated (24)). Second, what are the differences between the subsp. *xolocotziana* and its putative parents? For this question, an ANOVA was carried out considering a total of 73 individuals (subsp. *xolocotziana* (22), *A*. *mexicana* encouraged (29), and *A*. *palmeri* (22)).

In all cases, normality and homogeneity, Shapiro-Wilk and Levene, respectively, were determined prior to the analyses. Tukey’s post hoc test was used when necessary. When the variables did not meet the assumptions of normality and homogeneity, a Kruskal-Wallis test was used.

### Phytochemical evidence

#### Essential oil extraction

Once the morpho-physiological evaluation of the plants was completed, the essential oil was extracted from the aerial parts of the plants (stem, leaves, and inflorescence) for each population using hydrodistillation. Fifteen grams of pulverized plant material were extracted in 250 ml of distilled water to attain a final volume of 90 ml of an emulsion of essential oil and water. The essential oil was obtained by partition with ethyl acetate (1:1). The organic phase was recovered and concentrated under reduced pressure at a maximum temperature of 40 °C resulting in a final volume of 2 ml. The essential oil was stored in amber vials and kept refrigerated (4 °C) for further chemical profile analysis.

#### Thin layer chromatography

The chemical profile of the essential oil from each population was obtained by means of a thin layer chromatography (TLC); five main aromatic compounds were reported for “toronjil” ethnobotanical complex: estragole, geraniol, linalool, menthone, and pulegone [40, 53]. The analytical conditions of the TLC were the following: the stationary phase consists in silica gel chromatoplates (TLC silica gel 60 F_254_; Merck), the mobile phase was toluene–ethyl acetate (95:5), UV light (254 and 360 nm), and anisaldehyde reagent derivatization [54]. For each compound analyzed, the retention factor (*R*_f_) was calculated. Subsequently, the absence or presence of the five standards mentioned for each population was scored. The comparison was made by management categories.

## Results

### Ethnobotanical evidence

#### Use

The 80% of the people interviewed mention that the infusion combining *A*. *mexicana* and the subsp. *xolocotziana* is drunk to treat principally (75%) gastrointestinal, menstrual, and nerve pains as well as to combat “coraje” (an intense anger or disgust said to be an emotion expressed by great irritability) and secondarily (60%) in cases of “susto” or “espanto” (an ailment of cultural affiliation generated from an impression or deep fear, whose physical characteristics are: sunken eyes and yellow irises, paleness, loss of hunger, exhaustion, insomnia or drowsiness, and anxiety) [39]. Along with drinking the infusion, one is bathed nightly in a tub of hot water containing the “toronjiles,” until one is relaxed. In third place (40%), *A*. *mexicana* in form of poultices and infusions is recommended to alleviate pain generated by contusions. For treating these ailments, the people preferred using *A*. *mexicana* and the subsp. *xolocotziana* instead of árnica (*Heterotheca inuloides* Cass.) which is similarly employed.

### Traditional knowledge and selection criteria

Eighty percent of the people interviewed recognized two categories of *A*. *mexicana*: “toronjil morado de monte” (purple wild hyssop*)* and “toronjil morado de casa” (purple house hyssop) referring to the encouraged and cultivated populations, respectively. They differentiated the two based mainly on organoleptic (60%), floral (30%), and vegetative (10%) characteristics. The purple wild hyssop has a smell and taste of hyssop, that is, with a menthol aroma and with pale purple flowers, while the purple house hyssop has a strongly aniseed, sweet taste, and smell, with a more intense color of the leaves and flowers more intense than that of the wild one. The purple house hyssop is preferred over the wild to treat the ailments described above having greater consumption and effectiveness when the plants are in the floral stage.

Hundred percent of the informants referred to the subsp. *xolocotziana* as “toronjil blanco de casa” (white house hyssop) and mentions that this plant does not occur in the wild. They differentiate it from other hyssops mainly for their floral (70%), organoleptic (60%), and vegetative (30%) characteristics. They distinguish it by its flavor and smell of menthol, white flowers, olive-green leaves, and a stout rhizome.

The 80% of the people interviewed mentioned that purple and white house hyssops are highly valued for their medicinal effectiveness when consumed together as an infusion, having greater effectiveness when plants are in the floral stage and consumed in fresh. However, 50% also use them in the form of vegetative shoot or as shade-dried plants.

### Management

#### Encouraged populations of *A*. *mexicana*

All the people interviewed in San Pablo Oztotepec recognized that plants of purple wild hyssop tend to grow in disturbed habitats near “milpas,” traditional agricultural fields. The plants are valued for their medicinal and ornamental use and increase in density in response to weeding so as to remove other plants compete with them. At the time of harvesting these plants, people only cut the aerial part, leaving the rhizome so that it can produce new shoots during the next growing season. Another practice is that not all plants are harvested at the flowering time, thus leaving some inflorescences to produce seeds that subsequently fall to the ground and germinate.

#### Cultivated populations of *A*. *mexicana* and *A*. *m.* subsp. *xolocotziana*

*Agastache mexicana* and its subsp. *xolocotziana* (purple and white house hyssop, respectively) are widely cultivated in the town of Santiago Mamalhuazuca. Propagation is mainly done through the rhizome. However, 30% of the informants mention that they produce seedlings to introduce plants that are hardier and more resistant to environmental variations. However, in the case of white house hyssop, they mention that “few seeds germinate” and of those, they select the most vigorous plants to transplant although not all survive.

The interviewees of Santiago Mamalhuazuca mentioned that the white house hyssop is “chiquión” (that it does not withstand extreme changes in temperature and humidity) while the purple house hyssop is more resistant. The inhabitants of this town also cultivate other medicinal plants in their fields, such as fennel (*Foeniculum vulgare* Mill.), borage (*Borago officinalis* L.), rue (*Ruta chalepensis* L.), gordolobo (*Gnaphalium* spp.), pericón (*Tagetes lucida*), and epazote (*Dysphania graveolens*), that are grown in mosaic. Each year, they alternate the crops so as to favor the vigorous plants. The cultivation of purple and white hyssops begins in April so as to harvest the aerial part of the plants (stem and inflorescence) in November. The rhizome sprouts stems, thus allowing another harvest in the next month of February. The first harvest produces a greater quantity of plants than the second one, resulting in a higher sale price in February.

In Santiago Mamalhuazuca, the purple and white house hyssops are grown in greater quantity than other medicinal plants, such as pericón, rue, etc. The major sale of bundles and consumption (in mixture) occur when the plants are in the floral stage.

In the mixed bundles, the greater portion consists of the house purple hyssop rather than white hyssop. The people explain that the latter “gives less,” that is that the plants are smaller and produced in lesser quantities, especially when drastic climatic changes occur during the growing season. Based upon response to inquiries about provenance of cultivated germplasm (that is inherited among parents, siblings, and extended families), it is estimated that they have been cultivating the two Mexican hyssops for approximately 100 years.

### Morpho-physiological evidence

#### Discontinuities in the pattern of total variation and the clustering pattern

**Fig. 2:**
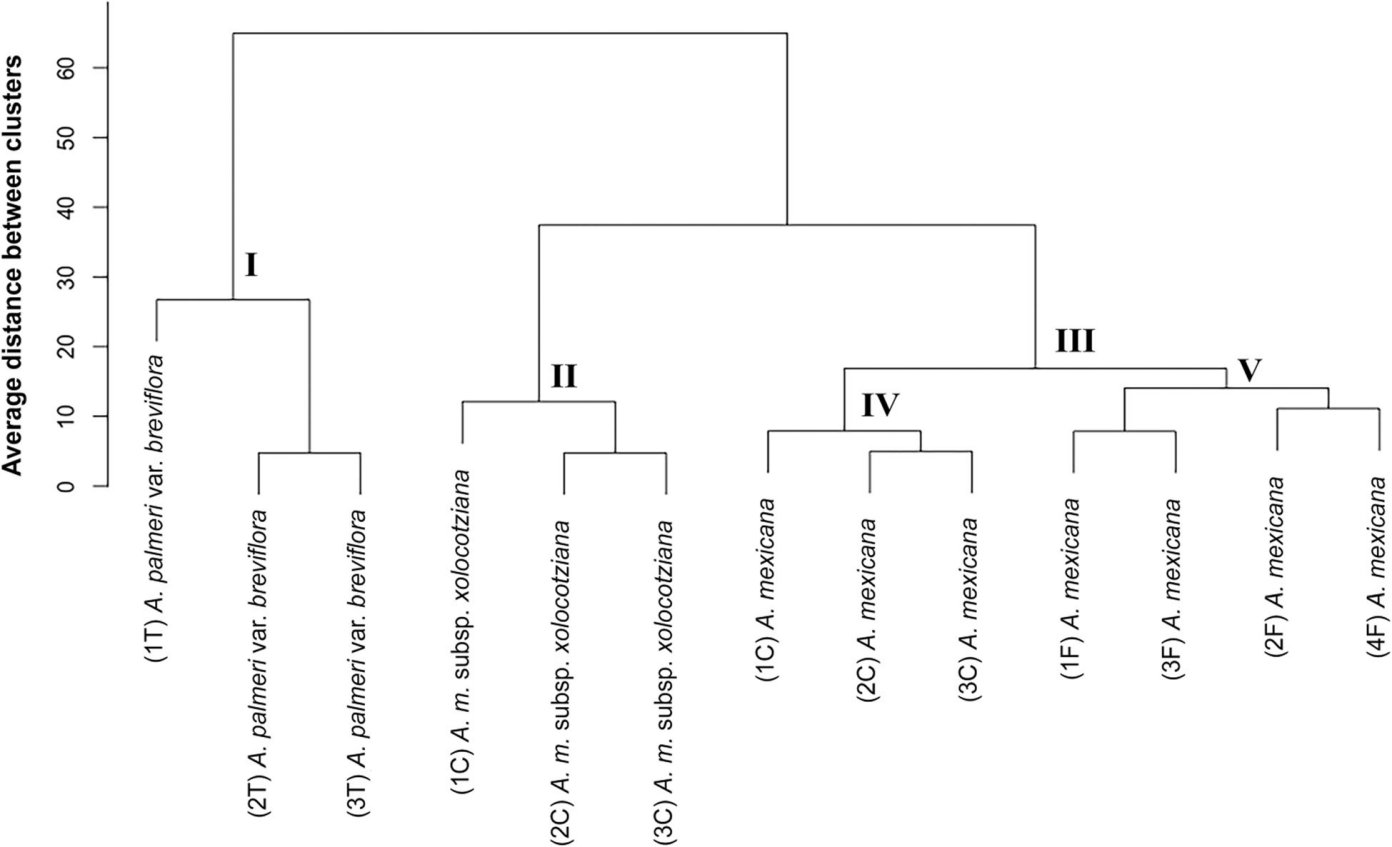
UPGMA dendrogram of the evaluated populations of *A*. *mexicana* and *A*. *palmeri*. F encouraged, C cultivated, and T tolerated

The evaluation of the 21 morphological characters in the 13 populations that include a total of 97 individuals reflects, in the UPGMA cluster analysis (Fig. 2), three groups: in the first group (I) are the three populations of *A*. *palmeri*, in the second (II) the three of the subsp. *xolocotziana*, while in the third (III) are the seven studied populations of *A*. *mexicana*. In this last group, two subgroups are observed, one that includes the four encouraged populations (V) and the other (IV) with the three cultivated.

**Fig. 3:**
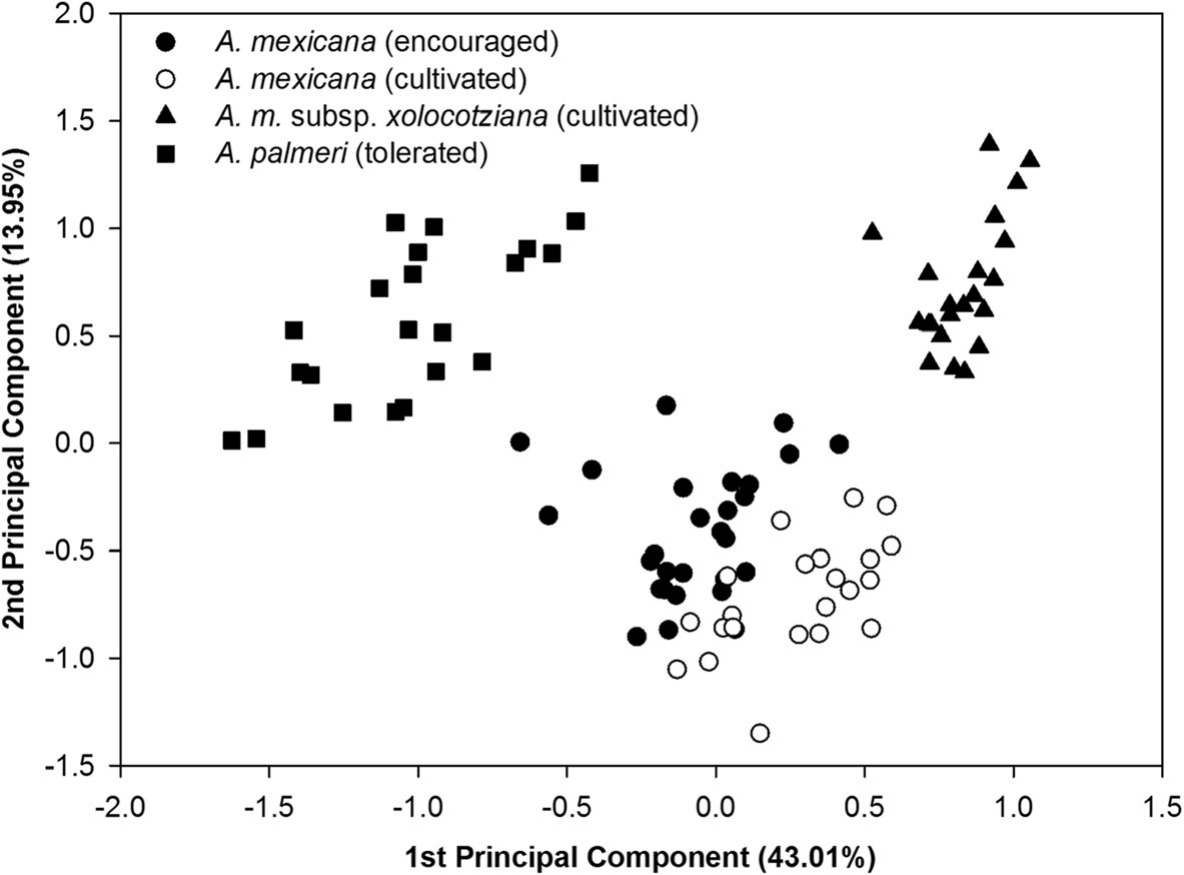
Graph of the first and second principal component derived from the evaluation of 20 morphological characters in the 97 individuals evaluated. Table 2 shows the vectors with the highest weight in each component

The dispersion of the individuals in the PCA chart (Fig. 3) supports the grouping of the previous analysis. In the upper left quadrant of Fig. 3 are the individuals of *A*. *palmeri*. In the lower center are the individuals of *A*. *mexicana* (where the cultivated plants of this taxon tend to separate from those encouraged population). Finally, the subsp. *xolocotziana* is segregated in the upper right quadrant. The first two components explain 43.01 and 13.95%, respectively, of the total variance of the individuals evaluated.

Considering the characters with greater weight in the first component, the cultivated plants of both *A*. *mexicana* and the subsp. *xolocotziana* have larger size (length) in structures related to flowers (style (0.312), upper and lower stamens (0.314 and 0.313, respectively), tube (0.281), and flower (0.314)), and greater seed length. In addition, the subsp. *xolocotziana* displays a smaller number of teeth in the margin of the leaf (− 0.252), a smaller number of flowers (− 0.251), and smaller plants (− 0.244) when compared with the *A*. *mexicana* and encouraged plants tolerated from *A*. *palmeri*.

The characters with greater weight in the second component show that the subsp. *xolocotziana* have a smaller number of nodes in the stem (− 0.457), differentiation in the color of the flower (0.362/white flowers), and a greater length in the rhizome (0.325) when compared to *A*. *mexicana* and *A*. *palmeri* (Fig. 3, Table 2).

#### Significant differences and trends in domestication of *A*. *mexicana* (encouraged and cultivated plants)

When comparing the encouraged and cultivated plants of *A*. *mexicana*, significant differences (*P* < 0.05) were found in 12 of the 20 characters evaluated. Table 3 shows the trends of domestication of *A*. *mexicana*.

**Table 3:** Averages and standard error of 12 characters that presented significant differences when comparing categories 1 (= encouraged) and 2 (= cultivated) in *A*. *mexicana*. Domestication trends that indicate these characters are presented

Characters		Domestication trend	Category	Mean/SE	Student *T*	*P*
Floral	Corolla color	More pigmented flowers	1	133.3 ± 1.72	4.28	0.000*
			2	120.1 ± 2.66		
	Inflorescence length	–	1	19.73 ± 1.91	3.02	0.003**
			2	12.15 ± 1.48		
	Style length	Longest style, gigantism	1	3.49 ± 0.05	3.58	0.000*
			2	3.74 ± 0.03		
	Length of lower stamens	Longer stamens, gigantism	1	2.74 ± 0.05	5.57	0.000*
			2	3.13 ± 0.03		
	Length of upper stamens		1	3.08 ± 0.06	5.06	0.000*
			2	3.47 ± 0.04		
	Flower tube length	Longest flower tube, gigantism	1	2.24 ± 0.04	5.47	0.000*
			2	2.59 ± 0.03		
	Flower length	Larger flowers, gigantism	1	2.74 ± 0.05	7.61	0.000*
			2	3.21 ± 0.02		
Seeds	Seed length	Longer and wider seeds, gigantism	1	1.8 ± 0.02	5.76	0.000*
			2	2.04 ± 0.03		
	Seed diameter		1	0.87 ± 0.02	4.09	0.000*
			2	1.02 ± 0.02		
Vegetative	Total height	-	1	98.83 ± 3.59	1.63	0.000*
			2	90.53 ± 3.48		
	Leaf area	Larger leaves, gigantism	1	7.74 ± 0.29	2.47	0.016***
			2	8.92 ± 0.38		
	Number of rhizome nodes	More nodes in the rhizome, greater asexual reproductive capacity	1	1.86 ± 0.12	2.69	0.009**
			2	2.5 ± 0.20		

* Significant level 0.001, ** significant level 0.01 and *** significant level 0.05

Five of these characters are related to reproductive structures. In cultivated plants, these characters suggest being related to gigantism in flowers and their greater pigmentation in them. With respect to the seeds, those of the cultivated form are larger than those of the encouraged form. These characteristics are very important in anthropocentric terms. Contrary to expectations, the cultivated Mexican hyssop has shorter inflorescences than the encouraged form.

Likewise, there are significant differences in two of three vegetative characters. The cultivated plants have larger leaves and a greater number of nodes in the rhizome than those of the encouraged plants. The differentiation in the leaves is related to a gigantism in the parts used. The difference in the rhizome suggests selection favoring greater asexual reproduction. However, total plant height of the cultivated Mexican hyssop is lower than that of the encouraged form.

#### Significant differences and trends in domestication in *A*. *m*. subsp. *xolocotziana*

When comparing the subsp. *xolocotziana* with its putative parents, *A*. *mexicana* (encouraged) and *A*. *palmeri*, 19 characters were found that differ significantly from the 21 characters evaluated in the study (Table 4).

**Table 4:** Means and standard error of 19 characters that presented significant differences when comparing the plants of (1) *A. m.* subsp. *xolocotziana* with (2) *A*. *mexicana* (encouraged) and (3) *A*. *palmeri*. Domestication trends of the subsp. *xolocotziana* are shown

Characters		Trend	Domestication	Category	Mean/SE	F/*X*^2^^	*P*
Floral	Corolla color	White	White flowers	1	193.6 ± 15^a^	403.7	0.000
				2	133.3 ± 1.72^b^		
				3	146.01 ± 1.2^c^		
	Style length	Longer style	Gigantism in flowers and correlated structures	1	4.08 ± 0.04^a^	59.0	0.000
				2	3.49 ± 0.05^b^		
				3	1.62 ± 0.01^c^		
	Length of lower stamens	Longer stamens		1	3.22 ± 0.04^a^	58.6^	0.000
				2	2.74 ± 0.05^b^		
				3	1.34 ± 0.01^c^		
	Length of upper stamens			1	3.6 ± 0.05^a^	58.6^	0.000
				2	3.08 ± 0.06^b^		
				3	1.53 ± 0.02^c^		
	Flower tube length	Longer tube		1	2.43 ± 0.07^a^	48.9^	0.000
				2	2.24 ± 0.04^b^		
				3	1.10 ± 0.02^c^		
	Flower length	Largest flower		1	3.26 ± 0.03^a^	60.0^	0.000
				2	2.74 ± 0.05^b^		
				3	1.52 ± 0.02^c^		
	Inflorescence length	Shorter inflorescence length	–	1	10.88 ± 1.07^b^	8.3^	0.01*
				2	19.73 ± 1.91^a^		
				3	16.45 ± 2.22^ab^		
	Number of nodes in the inflorescence	Smaller number of nodes	–	1	4.63 ± 0.24^b^	12.7^	0.000
				2	4.89 ± 0.3^b^		
				3	7.90 ± 0.73^a^		
	Number of flowers produced	Fewer flowers produced	–	1	46 ± 4.14^c^	18.3^	0.000
				2	86.2 ± 10^b^		
				3	185.6 ± 29^c^		
Seeds	Seed length	Longer and wider seeds	Seed in gigantism	1	2.08 ± 0.02^a^	84.68	0.000
				2	1.8 ±0.02^b^		
				3	1.5 ± 0.02^c^		
	Seed diameter			1	1.08 ± 0.03^a^	24.14	0.000
				2	0.8 ± 0.02^b^		
				3	0.86 ± 0.01^b^		
	Germination	Low percentage	Less sexual reproduction	1	30^b^	118	0.000
				2	90^a^		
				3	90^a^		
				3	111.02 ± 5.2^a^		
	Number of nodes in the stem	Smaller stem nodes	–	1	11 ± 0.27^b^	20.94^	0.000
				2	13.75 ± 0.31^a^		
				3	13.04 ± 0.72^a^		
	Leaf color	Less pigmentation in the leaf	Differentiation in leaf color	1	67.73 ± 1.03^a^	4.13	0.02*
				2	63.14 ± 2.7^ab^		
				3	59.04 ± 1.04^b^		
	Leaf area	Smaller leaf area	–	1	6.59 ± 0.32^a^	21.35	0.000
				2	7.74 ± 0.29^a^		
				3	10.03 ± 0.47^b^		
	Number of teeth on the leaf	Less teeth	–	1	11 ± 0.27^b^	35.54	0.000
				2	13.63 ± 0.38^a^		
				3	16.17 ± 0.53^a^		
	Rhizome Length	Longest rhizome with greater number of nodes	Gigantism in rhizome, greater asexual reproductive capacity	1	3.96 ± 0.28^a^	32.54	0.000
	Number of rhizome nodes			2	2.08 ± 0.23^b^		
				3	2.3 ± 0.21^b^		
				1	4.72 ± 0.26^a^	79.16	0.000
				2	2.51 ± 0.20^b^		
				3	1.72 ± 0.16^c^		

Different letters indicate significant differences. Significant level 0.001, * significant level 0.05

All the reproductive characters evaluated had significant differences. They indicate that the subsp. *xolocotziana* has white flowers (in contrast to the pigmented flowers), and larger corolla, although fewer flowers per inflorescence. Flower size is a human-selected feature. The seeds of subsp. *xolocotziana* are larger; however, they have a low germination percentage. The seed gigantism may be a pleiotropic effect that is related to flower size.

Seven vegetative characters differed significantly. The plants of the subsp. *xolocotziana* are shorter, with smaller leaves and fewer teeth in comparisons to *A*. *mexicana* and *A*. *palmeri*. Very significant differences are found in the rhizome. The longer rhizome with more nodes affords greater asexual reproductive capacity. This feature is directly related to its management mainly through vegetative propagation.

### Phytochemical evidence

Comparison of the chemical profiles of essential oils between the encouraged and cultivated plants of *A*. *mexicana* (Table 5) reveals that the former only present geraniol and pulegone, while the cultivated ones contain the five compounds evaluated: estragole, linalool, menthone plus the two cited above. All five compounds are present in the subsp. *xolocotziana* plants. In the case of *A*. *palmeri*, only three of them (geraniol, menthone, and pulegone) are registered (Table 5).

**Table 5:** Compounds, taxa, and management category considered in the study. (+) presence or (−) absence of the compounds

Compounds	*A*. *mexicana*		*A.m.* subsp. *xolocotziana*	*A*. *palmeri*
	Cultivated	Encouraged	Cultivated	Tolerated
Estragole	+	−	+	−
Geraniol	+	+	+	+
Linalool	+	−	+	−
Menthone	+	−	+	+
Pulegone	+	+	+	+

## Discussion

### Domestication of *Agastache*

This study presents ethnobotanical, morpho-physiological, and phytochemical evidence about the domestication processes of *A*. *mexicana* and *A*. *m.* subsp. *xolocotziana*. On the one hand, this information shows a differentiation between the encouraged and cultivated populations of *A*. *mexicana* along the domestication gradient. On the other hand, subsp. *xolocotziana* diverged from one of its putative parents of hybridization (Table 6).

**Table 6:** Domestication indicators in *A*. *mexicana* and *A. m.* subsp. *xolocotziana*

Evidences	Domestication Indicator	
	*A*. *mexicana*	*A.m.* subsp. *xolocotziana*
Ethnobotany: traditional knowledge and selection criteria according to human use and management:		
Traditional recognition	♦♦♦♦	♦♦♦♦
Plants known in cultivationas *house plants*	♦♦♦♦	♦♦♦♦
Distinguished by smell and taste	♦♦♦	♦♦♦
Morpho-physiologically distinguished	♦♦	♦♦♦♦
Preferred to treat ailments	♦♦♦♦	♦♦♦♦
Mainly vegetative propagation	♦♦♦♦	♦♦♦♦
Morpho-physiological:		
Significant differences	Yes	Yes
Gigantism in used parts	Yes	Yes
Gigantism in correlated structures	Yes	Yes
Phytochemicals:		
Differentiation in the composition of compounds in the essential oil	Yes	Yes
Differentiation related to human use and management	Yes	Yes

The symbols represent the portion of respondents who positively associated each factor: ♦♦ = 40%; ♦♦♦ = 60%; ♦♦♦♦ ≥ 80%

Organoleptic properties are fundamental in the plant domestication. Ankli et al. (1999b) and Brett and Heinrich (1998) reported that the organoleptic characteristics (mainly the aroma) of the plants are an important factor to determine them as medicinal, using them to define the relief ailment [18, 49]. Our work illustrates how these characteristics can also be fundamental in the process of domestication of aromatic medicinal plants, particularly in the hyssops. The people interviewed (Ozumba and Milpa Alta) recognize in *A*. *mexicana* an organoleptic differentiation between the plants encouraged, with a mild smell and flavor, and those cultivated, with an odor and sweet anise taste, relating the latter with greater effectiveness to alleviate different ailments, including gastrointestinal. At the same time, this is reflected in the phytochemical analysis where the cultivated hyssop has five aromatic compounds compared with the encouraged hyssop with only three aromatic compounds available (Table 5).

The morpho-physiological evidence indicates significant differences in floral, seed, and vegetative characteristics between the encouraged and cultivated plants of *A*. *mexicana* (Table 3). Flower size and corolla pigmentation were larger and more intense, respectively, in the cultivated populations. These characters are important selection criteria because the floral stage of *Agastache* is preferred state for consumption and is considered most effective as herbal remedy; hence, the cultivated category is the most appreciated.

Seed size was also larger in cultivated plants than in encouraged *A*. *mexicana* plants. However, ethnobotanical and physiological data show that this character is not directly selected. The interviewees mentioned that generally “all seeds germinate” and the physiological evaluation shows that there are no significant differences in the percentage of germination of the same; hence, the gigantism of this character is probably linked to the size of other related structures, possibly the largest size of the flower in cultivated plants.

As for the vegetative characters, the cultivated *A*. *mexicana* have a larger leaf size, a feature readily selected visually as being a valued trait of biomass yield. These characteristics were also found in the incipient domestication of cultivated epazote, selected for consumption as a condiment and as a medicinal herb [22]. Also, a greater number of nodes are found in the rhizome of the cultivated plants than those of the encouraged *A*. *mexicana*. This feature is related to its management, since vegetative propagation is preferred. Also, the increase of meristematic sites on the rhizome produces more stems that can be harvest.

In addition, significant differences were found in the total height and in the size of the inflorescence, being greater in the encouraged than in the cultivated ones of *A*. *mexicana*, opposite to the expected result, considering that those characteristics are subject to selection. These results may be associated with management, taking into account that the people interviewed mentioned that the plants developed from seeds in their first flowering period, “they do not grow much.” However, as time passes, the rhizome is strengthened and produces taller plants with larger inflorescences than those encouraged, explaining why the cultivation of these plants is mainly asexual.

When combining the three classes of evidence obtained in *A*. *mexicana*, a domestication syndrome is observed that consists of an organoleptic differentiation (smell and taste “sweet aniseed”) related to a phytochemical differentiation, floral gigantism, intensification of pigmentation, seed gigantism, and rhizome gigantism (enabling greater asexual reproductive capacity).

With respect to the origin of subsp. *xolocotziana*, the ethnobotanical, morpho-physiological, and phytochemical results clearly distinguish it from its putative parents, *A*. *mexicana* and *A*. *palmeri*, reflecting a series of domestication trends (Table 6). First, the organoleptic characteristics (mild mentholated) are very important for the traditional recognition of the subsp. *xolocotziana* in plantlet stage, since in its floral stage its differentiation is also based on the white color of the flower. The phytochemical differentiation is manifested by the presence of all five aromatic compounds analyzed (Table 5).

In morpho-physiological terms, flower color of the subsp. *xolocotziana* (white) was different compared to *A*. *mexicana* (purple) and *A*. *palmeri* (pink). Obviously, this trait is the basis of the folk nomenclature as well as recognition for selection during the floral stage and at the same time makes the plants more attractive to the consumer (Table 4 and Table 6).

The larger floral structures (size of the corolla, tube, style, and stamens) of the subsp. *xolocotziana* compared to *A*. *mexicana* (encouraged) and *A*. *palmeri* indicate gigantism in the flower (Table 4). Gigantism in this structure was also found in the cultivated plants of *A*. *mexicana*. In both taxa, the flowers are subject to selection, since they are a very important character for their medicinal consumption, where they are used together to potentiate their calming effect, as well as recognition by consumers [13, 31, 40].

Contrary to expectations, inflorescences of subsp. *xolocotziana* are smaller size (due to fewer number of nodes and of flowers) compared to their putative parents (Table 4). Some vegetative characters, such as the total height and area of the leaf (linked to a smaller number of teeth on the leaf) were also found to be reduced in this subspecies. Probably, as in the case of cultivated plants of *A*. *mexicana*, this is due to the plants being produced by seeds that reached only their first flowering period under experimental conditions. However, the people interviewed mention that of the harvest of subsp. *xolocotziana* is less than that of cultivated *A*. *mexicana*. It may be that dwarfism in these plants may represent depression.

The seeds also present gigantism in the subsp. *xolocotziana;* however, they are partially sterile, given the low percentage of germination found (30%) and the ethnobotanical information registered. In this sense, the gigantism in this structure is not related to its viability but may be linked to the gigantism present in the flower.

The rhizome-related characters in this subspecies also show significant differences when compared to the other two taxa, indicating gigantism with its longer rhizomes with a greater number of nodes (Table 4). These features are directly related to its management. Not only do these rhizomes facilitate rapid, one-step vegetative planting but also permit greater production of harvestable stems. A similar result was observed in cultivated plants of *Manihot esculenta* in which there was a greater production of propagules compared to their wild relatives [55].

The domestication syndrome of the subsp. *xolocotziana* combines elements of organoleptic and phytochemical differentiation, floral gigantism, floral albinism, seed gigantism, and rhizome gigantism. This latter feature offsets the disadvantage of seed sterility so as to favor cloning of a novel, desirable form of *Agastache*.

The morpho-physiological evidence shows a divergence of *A*. *mexicana* (encouraged) toward the subsp. *xolocotziana* (Fig. 3). Two major explanations may account for this situation. On the one hand, inbreeding depression in the typical *A*. *mexicana* may have given rise to the subsp. *xolocotziana*. In the populations of plants where disruptive evolutionary force operates, divergence can produce populations with lower fitness, which is expressed in its reduced vigor and fertility [56, 57]. These particularities are reflected in this taxon with less vigorous structures, lower germination percentage, and lower number of flowers produced (Table 4).

On the other hand, Abbott et al. [58] (2013) report that hybrids may not be morphologically and genetically intermediate to their parents, since one parent is dominant that it is difficult to detect the other in the hybrid. In this sense, this work does not rule out the hypothesis of the possible hybrid origin of the subsp. *xolocotziana*, since possibly *A*. *mexicana* has a morphological and genetic dominance over this subspecies.

In phytochemical terms, hybrids usually present additive, shared, and new compounds [59]. Based on this, the subsp. *xolocotziana* shows additivity in the menthone compound, and two shared compounds geraniol and pulegon with the possible parents (Table 5). In addition, Estrada-Reyes et al. (2004) reported the presence of 27 new compounds in the subsp. *xolocotziana* when compared with *A*. *mexicana*. It should be noted that in our work it was not possible to detect more compounds by the method used (TLC). Another novel character in this taxon is the white color of the flower; this particularity for instance has been found in the flowers of strawberry hybrids (*Fragaria* sp.) [60]. Also, partially sterile seeds are a trait of plants with a hybrid origin [61].

In order to clarify the origin of subsp. *xolocotziana*, phytochemical (more detailed gern technique) and genetic (phylogenetic and population genetics) studies of plant populations studied will provide more specific information about these two hypotheses.

### Maintenance strategies of cultivated germplasm

As for the encouraged populations of *A*. *mexicana*, they are found only on the periphery of the milpa agroecosystem, because in these places the inhabitants foster, maintain, and select “toronjil” for domestic use (medicinal and esthetic). Our study did not locate wild populations of this taxon in sites where it grew in 1980s [29]. Hence, the traditional management that the inhabitants of Milpa Alta provide in their milpa is as an important conservation mechanism for the permanence of this species.

In addition, the introduction of plants originating from seeds of *A*. *mexicana* crops is an important management and conservation strategy for these populations, since it has been reported that this practice helps maintain and generate genetic variability in vegetatively propagated crops [55, 62, 63].

Our ethnobotanical research indicates that the subsp. *xolocotziana* has high sensitivity to extreme environmental changes. Considering that it is known only from cultivation of mostly clonal plants, and that it exhibits partially sterile seeds, favoring the most vigorous individuals produced from seeds is fundamental for the conservation of this taxon. Several studies have documented that this type of management in vegetatively propagated crops can significantly increase their genetic diversity, making crops more resistant to environmental changes, such as agaves (*Agave angustifolia* Haw.) and cassava (*Manihot esculenta* Crantz) [55, 63–65].

## Conclusions

The evidence obtained indicates two divergent evolutionary processes under domestication. First, populations of *A*. *mexicana* growing in central Mexico are positioned medially along the domestication gradient and express elements of a domestication syndrome including organoleptic differentiation related to a phytochemical differentiation, floral gigantism, pigment intensification, seed gigantism, and rhizome gigantism. Second, *A. m.* ssp. *xolocotziana*, possibly originated by inbreeding depression or hybridization, demonstrates a syndrome with organoleptic and phytochemical differentiation, floral gigantism, albiflorism, seed gigantism, and rhizome gigantism. Each process has distinctive elements that make up its domestication syndrome which coincide with their importance in MTM to impart a calming effect in treating various ailments.

## Data Availability

Please contact first author for data requests.
